# Examining differences in cognitive and affective theory of mind between persons with high and low extent of somatic symptoms: an experimental study

**DOI:** 10.1186/s12888-017-1360-9

**Published:** 2017-05-30

**Authors:** Mira A. Preis, Dennis Golm, Birgit Kröner-Herwig, Antonia Barke

**Affiliations:** 1 Department of Clinical Psychology and Psychotherapy, Georg-Elias-Müller-Institute for Psychology, University of Göttingen, Göttingen, Germany; 2 Department of Child & Adolescent Psychiatry, Institute of Psychiatry, Psychology and Neuroscience, King’s College London, London, UK; 3 Department of Psychology, Division of Clinical Psychology and Psychotherapy, Philipps-University Marburg, Marburg, Germany

**Keywords:** Medically unexplained symptoms, Emotional awareness, Alexithymia, Theory of mind, Emotion recognition, Somatic symptoms

## Abstract

**Background:**

Medically unexplained somatic symptoms are common, associated with disability and strongly related to depression and anxiety disorders. One interesting, but to date rarely tested, hypothesis is that deficits in both theory of mind (ToM) and emotional awareness may undergird the phenomenon of somatization. This study sought to investigate whether or not differences in ToM functioning and self-reported emotional awareness are associated with somatic symptoms in a sample from the general population.

**Methods:**

The sample consisted of 50 healthy participants (37 females, 13 males) aged between 22 and 64 years (46.8 ± 11.7) of whom 29 reported a high extent of somatic symptoms (HSR), whereas 21 reported a low extent of somatic symptoms (LSR) based on the 30 highest and lowest percentiles of the Symptom List norms. The participants’ affective and cognitive ToM were assessed with two experimental paradigms by experimenters who were blind to the participants’ group membership. In addition, self-reports regarding emotional awareness, alexithymia, depressive and anxiety symptoms and current affect were collected.

**Results:**

In the experimental tasks, HSR showed lower affective ToM than LSR but the groups did not differ in cognitive ToM. Although HSR reported lower emotional awareness than LSR in the self-report measure, this group difference vanished when we controlled for anxiety and depression. Depression, anxiety, emotional awareness and alexithymia were correlated positively.

**Conclusions:**

The data supported the hypothesis that deficits in affective ToM are related to somatic symptoms. Neither cognitive ToM nor self-reported emotional awareness were associated with somatic symptoms. Self-reported emotional awareness, alexithymia and symptoms of depression and anxiety shared a considerable amount of variance.

**Electronic supplementary material:**

The online version of this article (doi:10.1186/s12888-017-1360-9) contains supplementary material, which is available to authorized users.

## Background

Medically unexplained somatic symptoms are common and give rise to high health care utilization resulting in substantial economic burden for the health care system [1–3]. They represent 22.9% of patients in general practice (12-months prevalence) [4]. An examination of the base rates of somatic symptoms in a representative sample showed that back pain, joint pain, pain in extremities, headache and abdominal symptoms (bloating or intolerance of several foods) as well as cardiovascular symptoms (palpitations) were the most frequently reported [5]. Extensive evidence has shown that the occurrence of somatic symptoms leads to disability [6], high frequencies of medical consultations [7] and is strongly related to depression and anxiety disorders [4, 8–12]. Somatic symptoms are more prevalent in patients with depression and/or anxiety disorders [8, 12] although causal relationships cannot be ascertained on the basis of the available evidence.

The former diagnoses of somatoform disorders in the Diagnostic and Statistical Manual of Mental Disorders (DSM, Fourth edition, text-revision; [13]) including somatization disorder, undifferentiated somatoform disorder, conversion disorder, pain disorder, hypochondriasis and body dysmorphic disorder were characterized by the occurrence of somatic symptoms that suggested, but could not be fully explained by, an underlying medical condition or the effect of a substance. As medically unexplained symptoms showed limited reliability, could accompany different medical conditions and reinforced the mind-body-dualism, the DSM-5 replaced ‘somatoform disorders’ with ‘somatic symptom disorder’ (SSD), characterized by somatic symptoms without the requirement that they be medically unexplained that are very distressing or result in significant disruption of functioning, as well as excessive and disproportionate thoughts, feelings and behaviors regarding those symptoms [14].

The development of somatic symptoms has been linked to reduced emotional awareness [15, 16] and deficits in the theory of mind [15–17]. According to an early model by Lane and Schwartz, emotional awareness – the capacity to perceive and describe one’s feelings – develops in five ascending stages (i) bodily sensations, (ii) action tendencies and/or global arousal, (iii) pervasive emotions, (iv) differentiated, attenuated emotions, (v) peak differentiation and blending of emotions [18]. In order to make sense of the experience of somatic symptoms one has to link them with an emotional state, a concept or thought or social interaction [17]. Subic-Wrana and colleagues proposed that decreased emotional awareness, which corresponds to lower stages in the model of Lane and Schwartz, may lead to the failure to experience affective arousal as feelings and instead process it as somatic symptoms [15, 19].

A related construct is alexithymia which literally means “lacking words for emotions” and which denotes the difficulty of identifying and describing one’s own emotional state [20]. This concept has proved fruitful in a number of contexts [21, 22], but it is still unclear whether persons with alexithymia mainly have a problem verbalizing emotions or identifying them in the first place [20].

Theory of mind (ToM) is defined as the ability to attribute mental states, such as desires, intentions and beliefs to oneself and other people [23] and is essential for social and behavioral functioning as it enables people to understand and predict behavior [24]. This multidimensional construct can be differentiated into two subcomponents: cognitive theory of mind, which describes a cognitive understanding of the difference between the speaker’s knowledge and that of the listener (knowledge about beliefs) and affective theory of mind, which additionally describes the empathic appreciation of the observed person’s emotional state (knowledge about emotions) [25]. Subic-Wrana and colleagues assessed emotional awareness and ToM in hospitalized patients with somatoform disorder and healthy controls and found significantly lower scores in both emotional awareness and ToM functioning in the patient group [15]. A similar result was found by Zunhammer and colleagues in somatoform pain patients [16]. According to them deficits in both emotional awareness and ToM functioning may underlie the phenomenon of somatization. This interesting conclusion, however, is based solely on the use of projective measures, as both the Frith-Happé-Animations Task (AT; [26]) measuring ToM, and the Levels of Emotional Awareness Scale (LEAS; [27]), evaluating emotional awareness, are of a projective nature beset with interpretative difficulties.

The main objective of the present study was to investigate with non-projective experimental tasks whether or not differences in ToM functioning and self-reported emotional awareness are associated with somatic symptoms. In accordance with the critique of medically unexplained symptoms that lead to the changes in the DSM, the present study focused on self-reported somatic symptoms regardless of whether they could be medically explained or not. By extending the work of Subic-Wrana and colleagues, who investigated patients with somatoform disorder [15], we investigated an analogue sample of healthy participants who reported a high (versus a low) extent of somatic symptoms. Analogue samples have several advantages: They afford more precise experimental control, often are more cost effective and allow disengaging factors that are an epiphenomenon of the disorder studied from factors that may present precursors [28]. We used two different paradigms that were developed specifically to assess affective and cognitive ToM and conducted the study with experimenters who were blind to the participants’ extent of somatic symptoms.

## Methods

### Design of the study

We used a quasi-experimental design with the between-subjects factor extent of physical symptoms, divided into two groups (see details below): participants who reported a high extent of somatic symptoms (HSR) and participants who reported a low extent of somatic symptoms (LSR). The experimenters were blind to the group membership of the participants throughout data collection and Faux Pas Recognition Test (FPRT) ratings (see below).

### Participants

#### Recruitment

Participants were recruited via newspaper advertisements in Göttingen and the surrounding area. The participants received 15 Euro for their participation. Following the procedure established by Bogaerts [29, 30], participants who indicated that they suffer from a pulmonary, cardiovascular, or neuromuscular disease, sarcoidosis, or diseases of the thyroid gland or the nervous system or gastrointestinal diseases were excluded from the study. In addition, non-native speakers of the German language were excluded in order to ensure that all participants were able to understand the instructions and the vignettes employed by the FPRT.

#### Sample characteristics

We studied an analogue sample of healthy participants who belonged to extreme groups regarding their amount of somatic symptoms (HSR, LSR). In a postal screening, 123 participants (90 female, 33 male) of the general population who had responded to the newspaper advertisements completed the Symptom List (SL; Beschwerden-Liste; [31]). Inclusion criteria for HSR were scores in the 30 highest and for the LSR scores in the 30 lowest percentiles according to the sex-specific SL norms. The inclusion criteria were fulfilled by 78 participants, of these, 28 were excluded due to the described exclusion criteria. The final sample consisted of *N* = 50 participants (37 females, 13 males) aged between 22 and 64 years (46.8 ± 11.7). Participants of the HSR group were older and reported more medical consultations and hospital stays than participants of the LSR group (for details see Table 1).

**Table 1:** Differences between the high and low symptom reporters (independent *t*-tests, Cohen’s *d*) in age, somatic symptoms (grouping variable), medical consultations and hospital stays

	*HSR (n = 29)*		*LSR (n = 21)*			*Differences*	
	*Mean*	*SD*	*Mean*	*SD*	*t (47)*	*p*	*d*
Age	49.76	10.66	42.71	12.01	2.19	.034	0.63
Somatic symptoms	29.10	8.70	4.69	2.50	12.46	<.001	3.57
Medical consultations (number last 12 months)	8.26	8.39	2.63	2.24	2.85	.001	0.85
Hospital stays (number last 12 months)	0.41	0.78	0.0	0.0	2.37	.022	0.68

*HSR* high symptom reporter, *LSR* low symptom reporter

### Experimental paradigms

#### Emotion recognition and affective theory of mind (ERaToM)

**Fig. 1:**
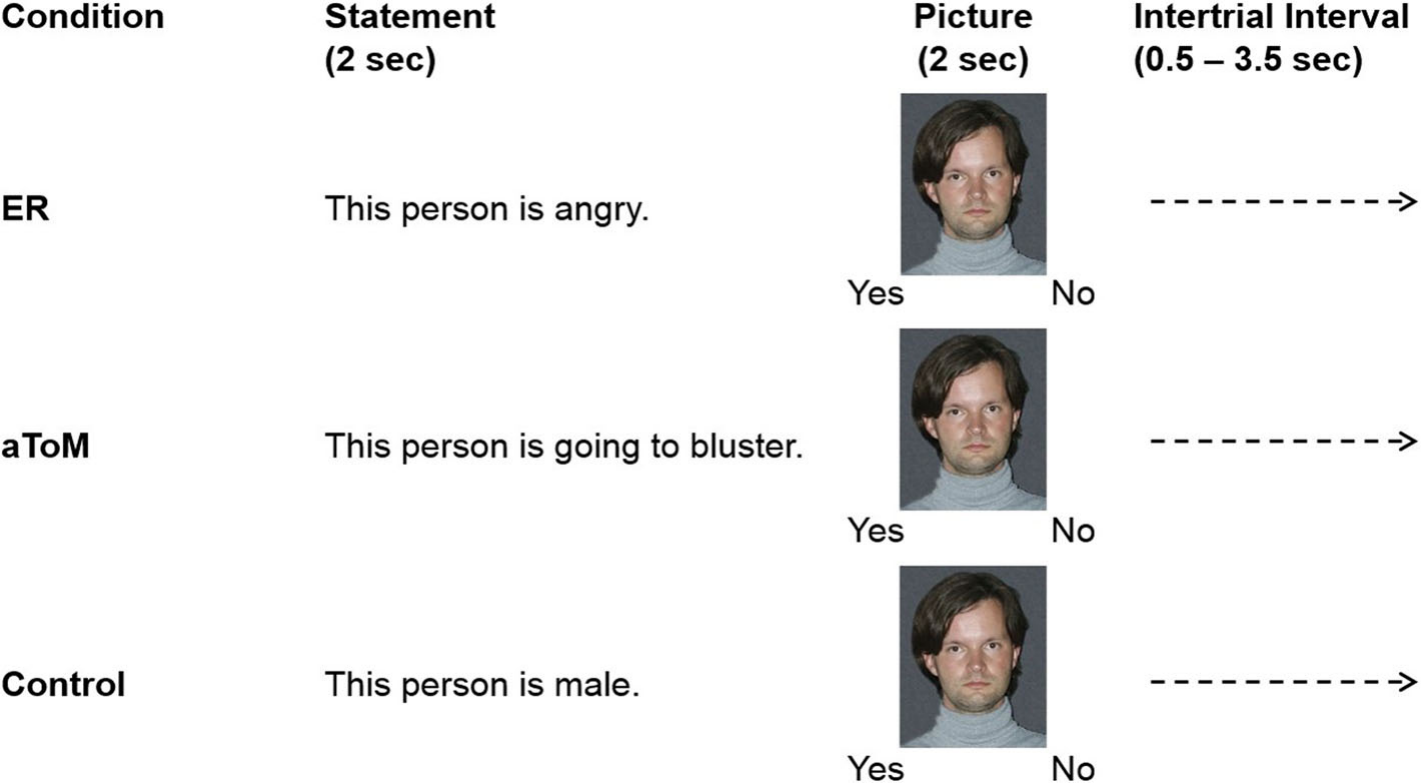
The three conditions of the emotion recognition and affective theory of mind (ERaToM). ER = emotion recognition; aToM = affective theory of mind

The computer-based paradigm developed by Mier and colleagues [32] measures the ability to recognize emotions from other peoples’ facial expressions (emotion recognition; ER) and to assign matching intentional states (affective theory of mind, aToM). The paradigm consists of three conditions: ER, aToM and a control condition. In each trial, a statement was displayed for 2 s, followed by a photograph of a face showing one of three different emotions (joy, anger, fear) or a neutral expression. The participants’ task was to evaluate whether or not the facial expression in the picture matched the preceding statement. Simultaneous with the picture, two possible answers were displayed (yes/no) and the participants had to press the corresponding button (yes/no). The displayed statements varied according to the condition (Fig. 1): For ER, the statement described one of three emotional states (This person is angry/afraid/happy). For aToM the statement described one of three emotional intentions/behaviors (This person is going to bluster/to run away/to cheer). The participant had to predict the action of the depicted person based on their facial expression of a specific emotion. The recognition of action intention can be interpreted as a basic process of ToM [33]. As all intentions in this task were driven by an affective state, Mier referred to them as aToM [32]. In the control condition the statement referred to a physical feature of the depicted person (This person is female/blond/older than 30). A total of 90 trials were displayed (30 trials per condition) in a pseudorandomized order.

An analysis of errors was carried out with regard to delayed answers, missing answers and twofold answers: if a participant did not answer within the given timeframe of two seconds (delayed answer) or not at all (missing answer), the trial was coded as an error; if a participant gave two answers (twofold answers), only the first answer was coded. After this process, the number of correct answers was calculated per condition (with a maximum of 30 per condition) and served as the operationalisation of ER and aToM, respectively.

#### Faux pas recognition test (FPRT)

**Fig. 2:**
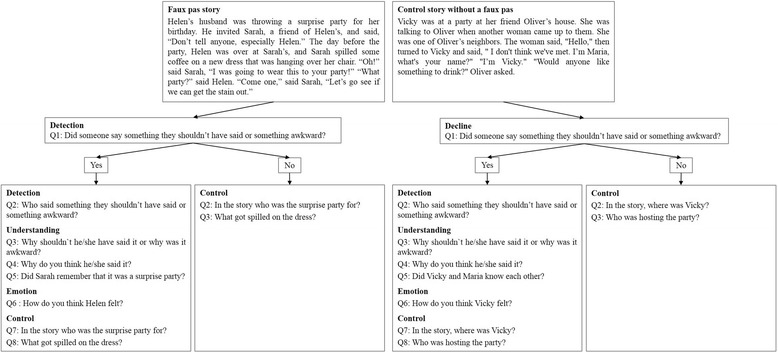
The two conditions (faux pas story, control story without a faux pas) and the questions asked in the Faux Pas Recognition Test (FPRT)

The FPRT assessed cognitive theory of mind (cToM); it was developed by Stone and colleagues [34]. Before using the German version of the FPRT translated by Ströbele [35], the translation was compared to the original by two bilingual speakers to ascertain the accuracy of the German version. It consists of 20 short stories, ten of which describe a situation in which a faux pas is committed, whereas the other ten are control stories without a faux pas. After each faux pas story, the experimenter asked the participant several questions. Two questions tested whether the participant detected the faux pas (detection). If the participant detected the faux pas, he or she was asked six further questions: three questions concerning a deeper understanding of the faux pas (understanding), one question testing whether the participant could place him or herself in the situation of the protagonist and guess his/her feelings (emotion) plus two control questions determining whether the participant generally understood the story (control). If a participant failed to detect the faux pas (no detection) he or she was directly asked the two control questions but no further questions. After a control story without a faux pas, the experimenter asked three questions: The first one determined whether the participant realised that there was no faux pas (decline) and two control questions concerned the general understanding of the story. If a participant wrongly assigned a faux pas to a control story, the experimenter still asked the six faux pas questions (detection, understanding of faux pas, emotion, and control) as if there had been a faux pas (see Figure 2).

In the faux pas stories, the participants’ scores on four scales were calculated: detection, understanding, emotion and general understanding (control questions). Each correct answer was counted as one point on that scale. In the control stories, two scales were calculated: decline and general understanding (control questions). If the participant correctly realised that there was no faux pas, two points were counted on the decline scale. If the participant wrongly assigned a faux pas to a control story, he or she received no points on the decline scale. The participants’ general understanding (control questions) of all stories was assessed independently from the other scales. Each correct answer was counted as one point on that scale even if he or she did not detect a faux pas or if he or she wrongly assigned a faux pas to a control story.

The stories were read to the participants, who also had a copy of the text so that they could simultaneously follow the story in the written version. The experimenters asked the questions orally and recorded the whole session with MP3 recorders. After the data acquisition, the two experimenters independently evaluated the recording using a pre-defined checklist. First, both experimenters separately evaluated the answers of two randomly selected participants and discussed diverging assessments afterwards. After agreeing and revising the checklist accordingly, the procedure was repeated with seven further participants. The interrater reliability was calculated from the independent ratings for the second seven cases and was *r* = .92. This was deemed sufficiently high for the experimenters to proceed separately from then onwards so that only one experimenter rated the answers of the remaining participants. (For the seven cases the divergences were resolved and the agreed values used for the analyses.)

### Psychometric instruments

#### Somatic symptoms

The SL [31] measures how much a person is affected by physical symptoms (e.g. shortness of breath, neck and shoulder pain). It has two parallel forms with 24 items each. We combined these to assess as many physical complaints as possible. The participants rated on a four-point scale from 0 (not at all) to 3 (strongly) how much he/she was affected by the complaint. Internal consistencies of both forms proved to be high in a healthy population (*α* = .93) as well as for a sample with psychological disorders (*α* = .94) [31]. Likewise, the internal consistency in the current sample was high (*α* = .97). As we included both parallel forms, the total value was divided by two (which resulted in a maximum achievable score of 72) in order to determine the percentiles on the basis of the norm sample [31]. In order to assign the participants to the two groups (HSR versus LSR), the sex-specific percentiles were used. Accordingly, women who reached a score ≤ 9 and men who reached a score ≤ 5 were classified as LSR (30 lowest percentiles). Women who reached a score ≥ 20 and men who reached a score ≥ 15 were classified as HSR (30 highest percentiles).

#### Emotional awareness

The Emotional Competence Questionnaire (ECQ; Emotionale Kompetenz Fragebogen; [36]) is a questionnaire that assesses the capacity to recognize, express and adequately cope with emotions. The ECQ contains four subscales: recognition and understanding of one’s own feelings (ES, e.g. 17. *I can easily recognize my feelings*), recognition and understanding of others’ feelings (EO, e.g. 6. *I can easily describe different emotional states of my friends*), regulation and control of one’s own feelings (RE, e.g. 19. *I can handle my feelings*), emotional expressivity (EX, e.g. 53. *I can easily put my feelings into words*). For the purpose of this study, the two subscales (ES and EO) concerned with emotional awareness were used. Internal consistencies for these subscales were good (*α*_ES_ = .88, *α*_EO_ = .91); exactly the same values were found for the present study [36].

#### Depression and anxiety

The German version of the Hospital Anxiety and Depression Scale (HADS) was used to assess anxiety and depression in the past week [37]. The HADS was designed for clinical populations suffering from somatic symptoms and consists of 7 items measuring anxiety (e.g. *I feel tense or overstrung)* and 7 items measuring depression (e.g. *I am happy*) on a 4-point scale. The internal consistencies for both subscales are satisfactory (Cronbach’s α = .80 for both Depression and Anxiety) and comparable to the present study (Depression subscale α = .81, Anxiety subscale α = .83) [37].

#### Positive and negative affect

The Positive and Negative Affect Schedule (PANAS; [38]) is a questionnaire to assess positive and negative affect. Each of the 20 items (10 assessing positive affect, e.g. *active, strong, proud, excited*, 10 assessing negative affect, e.g. *nervous, anxious, distressed, guilty*) are rated on a five-point rating scale from 1 (not at all) to 5 (very much). Internal consistencies of both scales are good (α_PA_ = .89, α_NA_ = .85) [38]. In the current sample, the internal consistencies were also good (α_PA_ = .91, α_NA_ = .90).

#### Alexithymia

The German version of the Toronto Alexithymia Scale (TAS-26; [39]) consists of three subscales: difficulty identifying feelings, difficulty describing feelings to others, and externally-oriented thinking. The 26 items are rated on a five-point rating scale from 1 (strongly disagree) to 5 (strongly agree) and can be used to calculate a sum score. Internal consistencies for the subscale difficulty identifying feelings (α = .84) and the total value (α = .81) are good in previous samples as well as in the present one (difficulty identifying feelings α = .90, total value α = .87) whereas internal consistencies for the subscales difficulty describing feelings (α = .69) and externally oriented thinking (α = .67) were less satisfactory [39]. In the present sample, the internal consistency for the subscale difficulty describing feelings was α = .76 and for the subscale externally-oriented thinking it was α = .52.

### Procedure

A written screening, which included the SL [31], demographic items (age, sex, highest education), the informed consent and some health-related questions (chronic diseases, use of medication, number of medical consultations and hospital stays in the last 12 months) was sent by post to potential participants. Participants who met the inclusion criteria (see Recruitment and Sample Characteristics) were invited to the laboratory part of the study which took place in the Georg-Elias-Müller Institute for Psychology in Göttingen and was conducted by two female experimenters, who were blind to the participants’ group membership. The study lasted approximately 90 min and was divided into two parts. In the first part, the participants’ aToM and cToM were assessed using the paradigms described (ERaToM, FPRT). In order to minimize sequence effects, the order of the two paradigms was balanced between participants and a break of 5 min between the two tasks was implemented. In the second part, the participants completed a set of questionnaires including the TAS-26 [39], the ECQ [36], the HADS [37] and the PANAS [38]. In the end, the participants were informed about the goals of the study and had the opportunity to ask questions.

### Statistical analyses

The differences between the two groups regarding the extent of somatic symptoms, emotional awareness, positive and negative affect, alexithymia, scores in the ERaToM and the FPRT were examined using independent analyses of covariance (ANCOVAs) with age and sex as covariates. The covariates were included because the sample size was rather small and variations in age and sex may influence the results. The groups showed an age difference (*M*_*HSR*_ = 49.76 ± 10.66; *M*_*LSR*_ = 42.71 ± 12.01; *t* (47) = 2.19, *p* = .034; Table 1). In addition, prior research suggested female superiority in theory of mind and the number of women was not equally distributed between the groups (even though the proportions did not differ significantly; HSR: 20 females, 9 males; LSR: 17 females, 4 males; *χ* ^*2*^ = 0.91, *p* = 0.34) [40]. For effect size, Eta squared is reported [41].

As the groups showed significant differences in anxiety/depression and positive and negative affect (see below), we conducted further exploratory analyses: ANCOVAs were employed to test whether the difference between the HSR and LSR in self-reported emotional awareness, affective ToM and alexithymia were maintained after controlling for the differences in anxiety/depression and negative and positive affect (the latter can be found in Additional file 1: Table S1). In order to analyze the relationships of the variables among each other, we calculated Pearson correlations. The significance level was set at *p* = 0.05.

## Results

### Correlations between relevant variables

The correlations between the variables were high (see Table 2), for example self-reported emotional awareness showed a strong negative correlation with anxiety/depression (*r* = −.53, *p* < .01), with positive and negative affect (*r* = .57, *p* < .01; *r* = −.57, *p* < .01) and with the sum score of the TAS-26 to measure alexithymia (*r* = −.76, *p* < .01).

**Table 2:** Correlations between the variables self-reported emotional awareness, anxiety/depression, positive and negative affect and alexithymia

	ECQ-ES	ECQ-EO	HADS	PANAS-pos	PANAS-neg
ECQ-EO	.45**				
HADS	−.53**	.16			
PANAS-pos	.57**	.07	−.66**		
PANAS-neg	−.57**	−.03	.79**	−.61**	
TAS-26	−.76**	−.37**	.49**	−.47**	.53**

*ECQ* Emotional Competence Questionnaire*, ES* emotion self*, EO* emotion other*, HADS* Hospital Anxiety and Depression Scale*, PANAS* Positive and Negative Affect Schedule*, neg* negative emotions*, pos* positive emotions*, TAS-26* Toronto Alexithymia Scale

**p < 0.01

### Group differences in ToM

#### Affective theory of mind (ERaToM)

Emotion recognition being a precondition of the aToM task, we calculated whether the groups differed in their ability to recognize the emotions of the displayed person’s facial expression (ER task) using an ANCOVA with age and sex as covariates. There were no group differences between HSR participants and LSR participants regarding the ER task (*F* (1, 45) = 2.40, *p* = 0.13); see Table 3 for means and standard deviations and Table 4 for the full ANCOVA results). The covariate age was significantly related to the participants’ ER score (*F*_*age*_ (1, 45) = 17.91, *p* < 0.001), whereas the covariate sex was not related to the participants’ ER score (*F*_*sex*_ (1, 45) = 3.07, *p* = 0.087). In order to evaluate whether HSR participants showed reduced aToM compared to LSR participants, we calculated an ANCOVA to compare the number of correct answers in the aToM task using age and sex as covariates. HSR participants showed significantly reduced aToM compared to LSR participants (*F* (1, 45) = 4.32, *p* = 0.043; Table 3, Table 4). The covariates were significantly related to the participants’ aToM (*F*_*age*_ (1, 45) = 8.65, *p* = 0.005; *F*_*sex*_ (1, 45) = 7.22, *p* = 0.010).

**Table 3:** Means and Standard Deviations of high and low symptom reporters concerning emotional awareness, the Emotion Recognition and affective Theory of Mind paradigm (ERaToM), the Faux Pas Recognition Test (FPRT), anxiety/depression, positive and negative affect and alexithymia

		*HSR (n = 29)*		*LSR (n = 21)*	
		*Mean*	*SD*	*Mean*	*SD*
Emotional Awareness					
ECQ-Emotion Self		3.44	0.68	3.89	0.62
ECQ-Emotion Other		3.87	0.42	3.80	0.45
ERaToM		*(n = 29)*		*(n = 20)*	
Emotion Recognition (max 30)		21.17	3.19	21.14	3.03
Affective Theory of Mind (max 30)		20.66	2.58	19.92	3.93
FPRT		*(n = 28)*		*(n = 21)*	
Faux pas stories					
Detection (max 20)		15.32	4.01	16.24	3.83
Understanding (max 30)		15.68	6.06	17.90	5.68
Emotion (max 10)		4.86	2.52	3.95	2.91
Control (max 20)		19.32	1.09	19.43	1.16
Control stories without a faux pas		*(n = 28)*		*(n = 21)*	
Decline (max 20)		18.07	2.28	18.48	1.99
Control (max 21)		20.11	1.26	20.24	0.99
Alexithymia					
TAS-26 sum score		2.52	0.61	2.12	0.42
Anxiety/Depression					
HADS sum score		14.86	7.69	7.05	4.51
Affect					
PANAS-pos		31.45	6.40	37.81	7.41
PANAS-neg		23.97	7.01	17.33	6.24

*The maximum achievable points concerning the ERaToM and FPRT are stated in brackets; due to technical problems, the log file of the ERaToM of one participant was corrupted and the data for the FPRT of another participant was missing. Thus, for both paradigms the sample size is N = 49; HSR* high symptom reporter*, LSR* low symptom reporter*, ECQ* Emotional Competence Questionnaire*, HADS* Hospital Anxiety and Depression Scale*, TAS-26* Toronto Alexithymia Scale*, PANAS* Positive and Negative Affect Schedule*, pos* positive affect*, neg* negative affect

**Table 4:** Differences between high and low symptom reporters (Analysis of Covariance with age and sex as covariates) in the Emotion Recognition and affective Theory of Mind paradigm (ERaToM)

Source	*df*	*MS*	*F*	*p*	*η* ^*2*^
Emotion Recognition					
Covariate age	1	124.41	17.91	< .001	0.262
Covariate sex	1	21.32	3.07	.087	0.045
Group	1	16.70	2.40	.128	0.035
Error	45	6.95			
Total	49				
Affective Theory of Mind					
Covariate age	1	67.92	8.65	.005	0.133
Covariate sex	1	56.66	7.22	.010	0.111
Group	1	33.93	4.32	.043	0.066
Error	45	7.85			
Total	49				

#### Cognitive theory of mind (FPRT)

In order to assess whether HSR participants showed reduced cToM compared to LSR participants, we performed independent ANCOVAs regarding the 6 scales of the FPRT (faux pas: detection, understanding, emotion, control; non faux pas: decline, control) using age and sex as covariates. No group differences were found (Table 3, Table 5).

**Table 5:** Differences between high and low symptom reporters (Analysis of Covariance with age and sex as covariates) in the Faux Pas Recognition Test (FPRT)

Source	*df*	*MS*	*F*	*p*	*η* ^*2*^
Faux pas stories					
Detection					
Covariate age	1	0.12	0.01	.931	0.000
Covariate sex	1	0.37	0.02	.881	0.000
Group	1	7.82	0.48	.490	0.011
Error	45	16.17			
Total	49				
Understanding					
Covariate age	1	33.51	0.95	.334	0.020
Covariate sex	1	20.20	0.58	.452	0.012
Group	1	23.49	0.67	.418	0.014
Error	45	35.161			
Total	49				
Emotion					
Covariate age	1	36.51	5.41	.025	0.100
Covariate sex	1	0.03	0.01	.946	0.000
Group	1	23.38	3.46	.069	0.064
Error	45	6.75			
Total	49				
Control Questions					
Covariate age	1	0.68	0.61	.441	0.011
Covariate sex	1	7.93	7.05	.011	0.134
Group	1	0.09	0.08	.780	0.001
Error	45	1.13			
Total	49				
Control stories without a faux pas					
Decline					
Covariate age	1	0.05	0.01	.919	0.000
Covariate sex	1	2.21	0.46	.501	0.010
Group	1	1.09	0.23	.637	0.005
Error	45	4.82			
Total	49				
Control Questions					
Covariate age	1	0.66	0.54	.468	0.011
Covariate sex	1	6.71	5.48	.024	0.107
Group	1	0.03	0.03	.868	0.001
Error	45	1.23			
Total	49				

### Group differences in self-reported emotional awareness

We calculated two ANCOVAs to test whether the HSR and the LSR groups differed with regard to the two subscales of the ECQ while using age and sex as covariates. HSR participants reported significantly lower emotional awareness of their own emotions than LSR participants (*F* (1, 46) = 7.33, *p* = 0.009; Table 6). The covariates were not related to the participants’ self-reported emotional awareness (*F*_*age*_ (1, 46) = 2.71, *p* = 0.11; *F*_*sex*_ (1, 46) = 0.12, *p* = 0.745).

**Table 6:** Differences between high and low symptom reporters (Analysis of Covariance with age and sex as covariates) in self-reported emotional awareness

Source	*df*	*MS*	*F*	*p*	*η* ^*2*^
ECQ-ES					
Covariate age	1	1.14	2.71	.106	0.048
Covariate sex	1	0.05	0.11	.745	0.002
Group	1	3.08	7.33	.009	0.131
Error	46	0.42			
Total	50				
ECQ-EO					
Covariate age	1	0.13	0.72	0.399	0.015
Covariate sex	1	0.42	2.30	0.136	0.047
Group	1	0.04	0.19	0.662	0.004
Error	46	0.18			
Total	50				

*ECQ* Emotional Competence Questionnaire*, ES* emotion self*, EO emotion other*

The groups did not differ with regard to the recognition of other’s emotions (*F* (1, 46) = 0.19, *p* = 0.662) and the covariates were not related to the participants’ recognition of other’s emotions (*F*_*age*_ (1, 46) = 0.72, *p* = 0.400; *F*_*sex*_ (1, 46) = 2.30, *p* = 0.136; Table 3, Table 6).

### Group differences in depression, anxiety, affect and alexithymia

In order to calculate whether the groups differed in depression, anxiety, affect or alexithymia ANCOVAs were calculated using age and sex as covariates. Compared to the LSR participants, HSR participants reported significantly higher depression and anxiety ratings (*F* (1, 46) = 18.44, *p* < 0.001), more negative and less positive affect (*F*_*positive affect*_ (1, 46) = 12.42, *p* = 0.001; *F*_*negative affect*_ (1, 46) = 14.14, *p* < 0.001) and higher alexithymia scores (*F* (1, 46) = 8.02, *p* = 0.007). Neither age nor sex were related to these variables (Table 3, Table 7).

**Table 7:** Differences between high and low symptom reporters (Analysis of Covariance with age and sex as covariates) in the anxiety/depression, positive and negative affect and alexithymia

Source	*df*	*MS*	*F*	*p*	*η* ^*2*^
Alexithymia (TAS-26 sum score)					
Covariate age	1	0.69	2.38	.130	0.042
Covariate sex	1	0.04	0.13	.718	0.002
Group	1	2.32	8.02	.007	0.142
Error	46	0.29			
Total	50				
Anxiety/Depression (HADS)					
Covariate age	1	71.16	1.64	.206	0.025
Covariate sex	1	0.18	0.00	.949	0.000
Group	1	798.91	18.44	< .001	0.279
Error	46	43.33			
Total	50				
Affect					
PANAS-pos					
Covariate age	1	110.27	2.38	.130	0.039
Covariate sex	1	1.36	0.03	.865	0.000
Group	1	575.44	12.42	.001	0.204
Error	46	46.33			
Total	50				
PANAS-neg					
Covariate age	1	133.31	3.06	.087	0.048
Covariate sex	1	16.07	0.37	.547	0.006
Group	1	616.47	14.14	< .001	0.222
Error	46	43.59			
Total	50				

*TAS-26* Toronto Alexithymia Scale*, HADS* Hospital Anxiety and Depression Scale*, PANAS* Positive and Negative Affect Schedule*, pos* positive affect*, neg* negative affect

### Exploratory analyses

#### No influence of anxiety and depression on group differences in aToM

An ANCOVA with the aToM score as dependent and group as independent variables with the HADS score in addition to age and sex as covariates showed significant group differences (*F* (1, 44) = 4.54, *p* = 0.039), with a higher aToM score in the LSR group than in the HSR group. The covariate HADS score was not related to the participants’ aToM score (*F* (1, 44) = 0.51, *p* = 0.481) while age and sex were related to the participants’ aToM (*F*_*age*_ (1, 44) = 9.04, *p* = 0.004; *F*_*sex*_ (1, 44) = 7.10, *p* = 0.011; Table 8).

**Table 8:** Differences between high and low symptom reporters (Analysis of Covariance with depression/anxiety, age and sex as covariates) in aToM (affective ToM), emotional awareness (Emotion Other) and alexithymia

Source	*df*	*MS*	*F*	*p*	*η* ^*2*^
Affective Theory of Mind (aToM)					
Covariate age	1	71.73	9.04	.004	0.139
Covariate sex	1	56.39	7.10	.011	0.109
Covariate HADS	1	4.01	0.51	.481	0.008
Group	1	36.05	4.54	.039	0.675
Error	44	7.94			
Total	49				
Emotional Awareness (Emotion Other)					
Covariate age	1	0.50	1.41	.241	0.025
Covariate sex	1	0.04	0.11	.745	0.002
Covariate HADS	1	3.40	9.58	.003	0.169
Group	1	0.25	0.70	.408	0.792
Error	45	0.36			
Total	50				
Alexithymia (TAS-26)					
Covariate age	1	0.32	1.26	.268	0.023
Covariate sex	1	0.03	0.13	.720	0.002
Covariate HADS	1	1.78	6.94	.012	0.127
Group	1	0.33	1.29	.263	0.824
Error	45	0.29			
Total	50				

*TAS-26* Toronto Alexithymia Scale*, HADS* Hospital Anxiety and Depression Scale

#### Influence of anxiety and depression on group differences in self-reported emotional awareness

An ANCOVA concerning the self-reported emotional awareness scores as dependent and group as independent variables with the HADS score in addition to age and sex as covariates showed no significant group differences (*F* (1, 45) = 0.70, *p* = 0.408). The covariate HADS score was significantly related to the participants’ self-reported emotional awareness (*F* (1, 47) = 9.58, *p* = 0.003) while age and sex were not related to the participants’ self-reported emotional awareness (*F*_*age*_ (1, 45) = 1.41, *p* = 0.241; *F*_*sex*_ (1, 45) = 0.11, *p* = 0.745; Table 8).

#### Influence of anxiety and depression on group differences in alexithymia

As the groups showed significant differences in anxiety and depression, we performed an ANCOVA examining the group differences in alexithymia to evaluate whether the group differences were maintained after controlling for the differences in anxiety and depression in addition to age and sex. The ANCOVA with alexithymia as a dependent and group as an independent variable and HADS score, age and sex as covariates showed no significant group differences (*F* (1, 45) = 1.29, *p* = 0.263). The covariate HADS was significantly related to the participants’ alexithymia scores (*F*(1, 45) = 6.94, *p* = 0.012) whereas the covariates age and sex showed no influence on the participants’ alexithymia scores (*F*_*age*_ (1, 45) = 1.26, *p* = 0.268; *F*_*sex*_ (1, 45) = 0.13, *p* = 0.720; Table 8).

## Discussion

The main objective of this study was to investigate whether or not the occurrence of somatic symptoms is associated with difficulties in emotional awareness and ToM functioning. Comparing healthy participants who reported a high versus a low extent of somatic symptoms while controlling for age and sex revealed differences in aToM but not in cToM. When controlling for the differences in anxiety and depression, there were no differences in self-reported emotional awareness between the groups.

### HSR and LSR participants differed in aToM but not in cToM functioning

As the multidimensional construct of ToM can be differentiated in two subcomponents (cToM and aToM), we used two corresponding tests – the FPRT and the ERaToM – which were specifically designed to assess these components, respectively [32, 34]. As a precondition for the aToM task, we also tested whether the groups differed in their ability to recognize the emotions from facial expressions and found no differences. There were no group differences between HSR participants compared to LSR participants regarding their cToM, but participants of the LSR group showed higher aToM than participants of the HSR group. This divergent result concerning aToM and cToM is in line with Stonnington and colleagues [17] who failed to find impaired cToM functioning in patients with somatoform pain disorders (as compared to healthy controls), but whose results also indicated reduced aToM functioning in their patient group. Subic-Wrana and colleagues [15] found reduced ToM functioning in patients with somatoform disorders when compared to healthy controls and Zunhammer and colleagues [16] reported the same for patients suffering from chronic somatoform pain. However, in the latter two studies, affective and cognitive ToM were not differentiated hindering a direct comparison of the results. With regard to aToM the current study is in line with their results and indicates the need for a differentiated approach that incorporates both facets of ToM. The current study extended previous results as it demonstrated that aToM differences can be observed not only by contrasting patient groups with healthy controls but such differences in functioning can be found already in an analogue sample. This is of particular importance as it may indicate that aToM deficits precede and, perhaps, promote the development of a somatic symptom disorder.

The fact that the high symptom reporters differed from the low symptom reporters in affective but not in cognitive ToM may also be rooted in different requirements of the tasks: whereas the ERaToM is a speeded computer based test, the Faux pas test allows more time and context information; both of which may help to solve the task. Possibly, existing slight deficits of the high symptom reporters could be compensated in the cognitive Faux pas test but not in the speeded ERaToM.

So far, studies that found impaired ToM in patients with somatoform disorders [15–17] assessed ToM with the AT. The AT is a projective measure that uses visual animations featuring two moving triangles in a framed background. The triangles’ movements can be interpreted as ‘interactions’ that ‘make sense’ if the observer considers that the triangles think, feel or intend (e.g. one triangle wants to surprise the other). The participants’ task is to describe what happens in the animation. The answers are then coded firstly with regard to the degree to which the participants interpreted the purpose of the actions of one triangle as influencing the mental state of the other triangle and how appropriately the script had been captured. We conducted the present study to broaden the empirical basis by including paradigms that assess ToM with ecologically more valid stimuli. Firstly, this allows excluding the participants’ verbal fluency as a confounding factor. In addition, and more crucially, as ToM refers to the ability to attribute mental states to *persons* (oneself and other people; [23]) it seemed desirable to assess this construct by testing whether people assign feelings to real persons who indeed showed emotional facial expressions rather than to geometrical figures, which in reality do not have any feelings at all. The fact that differences in aToM were found with a different experimental paradigm contributes to showing that the differences found may indeed be robust.

To sum up, the current results indicate a deficit in aToM in participants reporting a high amount of somatic symptoms and clearly highlight the need for future research that uses different and varied paradigms to assess the two components of ToM.

### HSR and LSR participants did not differ in self-reported emotional awareness after controlling for anxiety/depression

At first glance, HSR participants reported less emotional awareness regarding their own emotions than LSR participants, corresponding well with previous results [15, 16, 19]. However, self-reported emotional awareness was strongly correlated with symptoms of anxiety and depression. In line with previous research [8–10, 12], HSR participants reported higher anxiety and depression scores in the HADS [37] than LSR participants. For this reason, we explored the relationship between self-reported emotional awareness, somatic symptoms and depression/anxiety more closely and found that HSR participants did not differ in their awareness of their own emotions when we controlled for their elevated depression/anxiety scores. The same held true when we used alexithymia as a dependent variable and depression/anxiety as a control variable. Thus the observation that differences in self-reported emotional awareness are abolished when controlling for depression and anxiety, does not seem to be specific to the questionnaire used, but appears to represent a more robust finding. These results correspond with those by Stonnington [17] and Lane [20], but are in contrast with Subic-Wrana [15], who found significantly lower emotional awareness in patients with somatoform disorders when compared to healthy controls, but no relationship between depression and emotional awareness. However, in their paper, they did not report the respective statistical measures.

One possible explanation for the diverging results may lie in the investigated populations. In [15] and [16] the emotional awareness of hospitalized patients was compared with healthy controls, whereas in the current study the sample comprised healthy patients who differed in their extent of somatic symptoms. It is possible that the differences in emotional awareness may be too small to be detected in a healthy population. However, Stonnington [17] who investigated outpatients whose level of suffering might lie considerably above healthy participants but slightly below that of inpatients also failed to find impairments in the emotional awareness. Differences between hospitalized patients and healthy controls are not limited to somatic symptoms; hence it is also possible that other confounding factors may explain the group differences found in these studies.

### Strengths and limitations

A strength of this study lies in the robust methodical procedure: ToM was assessed by two different experimental paradigms that were specifically developed to assess the two subcomponents of ToM, were multimodal (computer tasks, text vignettes), required different sensory approaches (auditory, visual) and were conducted by experimenters who were blind to the participants’ extent of somatic symptoms. The sample was recruited from the general population of Göttingen and the surrounding area. In spite of the fact that we compared an analogue sample formed by using extreme groups with regard to their extent of somatic symptoms, the participants were all healthy, which decreases possible confounding factors. However, the fact that two key variables (sample and measures) differed from previous studies, limits the direct comparability of the results. The negative results concerning cToM and emotional awareness have to be interpreted in light of the limited sample size, which – although comparable to prior studies [15, 16] – would still benefit from replication in a larger sample.

## Conclusions

Comparing healthy participants who reported a high versus a low extent of somatic symptoms revealed differences in aToM but not in cToM. When controlling for the differences in anxiety and depression there were no differences in emotional awareness between the groups. In future research, anxiety and depression should be included as control variables when investigating emotional awareness. The results highlight the need for future studies that assess ToM and emotional awareness by means of paradigms with varying degrees of difficulties, diverging populations and larger samples in order to answer the question whether or not deficits in emotional awareness or ToM functioning may undergird the phenomenon of somatization.

## Additional file

Additional file 1: Table S1. Differences between high and low symptom reporters (Analysis of Covariance with affect, age and sex as covariates) in aToM (affective ToM), emotional awareness (Emotion Other) and alexithymia. (DOCX 17 kb)

## Abbreviations

ANCOVA: Analysis of covariance
aToM: Affective theory of mind
AT: Frith-Happé-Animations Task
cToM: Cognitive theory of mind
DSM: Diagnostic and Statistical Manual of Mental Disorders
ECQ: Emotional Competence Questionnaire
ERaToM: Emotion recognition and affective theory of mind
ER: Emotion recognition
EO: Recognition and understanding of others’ feelings
ES: Recognition and understanding of one’s own feelings
EX: Emotional expressivity
FPRT: Faux Pas Recognition Test
HADS: Hospital Anxiety and Depression Scale
HSR: High symptom reporter
LEAS: Levels of Emotional Awareness Scale
LSR: Low symptom reporter
PANAS: Positive and Negative Affect Schedule
RE: Regulation and control of one’s own feelings
SSD: Somatic symptom disorder
SL: Symptoms list
ToM: Theory of mind
TAS-26: Toronto Alexithymia Scale
