# Supplementary material for: Examining differences in cognitive and affective theory of mind between persons with high and low extent of somatic symptoms: an experimental study
Source: BMC Psychiatry. 2017 May 30;17:200. doi: 10.1186/s12888-017-1360-9 (PMC5450064; doi:10.1186/s12888-017-1360-9)
Supplement: Additional file 1: Table S1. — Differences between high and low symptom reporters (Analysis of Covariance with affect, age and sex as covariates) in aToM (affective ToM), emotional awareness (Emotion Other) and alexithymia. (DOCX 17 kb) [file 12888_2017_1360_MOESM1_ESM.docx]

**Additional file1: Table S1** Differences between high and low symptom reporters (Analysis of Covariance with affect, age and sex as covariates) in aToM (affective ToM), emotional awareness (Emotion Other) and alexithymia.

| **Source** | | ***df*** | ***MS*** | ***F*** | ***p*** | ***η^2^*** |
| --- | --- | --- | --- | --- | --- | --- |
| **Affective Theory of Mind (aToM)** | |  |  |  |  |  |
|  | **Covariate age** | 1 | 57.82 | 7.08 | .011 | 0.421 |
|  | **Covariate sex** | 1 | 58.26 | 7.13 | .011 | 0.424 |
|  | **Covariate PANAS-pos** | 1 | 0.18 | 0.02 | .884 | 0.001 |
|  | **Covariate PANAS-neg** | 1 | 1.94 | 0.24 | .628 | 0.014 |
|  | **Group** | 1 | 19.06 | 2.33 | .134 | 0.139 |
|  | **Error** | 43 | 8.17 |  |  |  |
|  | **Total** | 49 |  |  |  |  |
|  |  |  |  |  |  |  |
| **Emotional Awareness (Emotion Other)** | |  |  |  |  |  |
|  | **Covariate age** | 1 | 0.14 | 0.47 | .498 | 0.046 |
|  | **Covariate sex** | 1 | 0.00 | 0.01 | .918 | 0.001 |
|  | **Covariate PANAS-pos** | 1 | 1.64 | 5.33 | .026 | 0.522 |
|  | **Covariate PANAS-neg** | 1 | 1.31 | 4.24 | .045 | 0.416 |
|  | **Group** | 1 | 0.05 | 0.16 | .691 | 0.016 |
|  | **Error** | 44 | 0.31 |  |  |  |
|  | **Total** | 50 |  |  |  |  |
|  |  |  |  |  |  |  |
| **Alexithymia (TAS-26)** | |  |  |  |  |  |
|  | **Covariate age** | 1 | 0.13 | 0.53 | .470 | 0.075 |
|  | **Covariate sex** | 1 | 0.01 | 0.02 | .884 | 0.003 |
|  | **Covariate PANAS-pos** | 1 | 0.34 | 1.42 | .241 | 0.200 |
|  | **Covariate PANAS-neg** | 1 | 1.05 | 4.31 | .044 | 0.609 |
|  | **Group** | 1 | 0.20 | 0.80 | .375 | 0.114 |
|  | **Error** | 44 | 0.24 |  |  |  |
|  | **Total** | 50 |  |  |  |  |

*Note. TAS-26 = Toronto Alexithymia Scale; PANAS = Positive and Negative Affect Schedule, pos = positive affect, neg = negative affect.*
